# Hepatotoxicity Risk of Isoniazid in Patients with Autoimmune Rheumatic Diseases and Prior Liver Injury Due to Disease-Modifying Antirheumatic Drugs: A Single-Center Experience and Literature Review

**DOI:** 10.3390/jcm15020432

**Published:** 2026-01-06

**Authors:** Joy Selene Osorio-Chávez, Virginia Portilla González, Iván Ferraz-Amaro, Santos Castañeda, José Manuel Cifrián Martínez, Ricardo Blanco Alonso

**Affiliations:** 1 Pneumology, Hospital Universitario Marqués de Valdecilla, Valdecilla Santander Research Institute, Immunopathology Group (IDIVAL), 39008 Santander, Spain; joyselene.osorio@scsalud.es (J.S.O.-C.); jose.cifrian@scsalud.es (J.M.C.M.); 2 Rheumatology, Hospital Universitario Marqués de Valdecilla, Valdecilla Santander Research Institute, Immunopathology Group (IDIVAL), Avda. Valdecilla s/n, 39008 Santander, Spain; vportilla@humv.es; 3 Rheumatology, Hospital Universitario de Canarias, 38320 Santa Cruz de Tenerife, Spain; iferrazamaro@hotmail.com; 4 Rheumatology, Hospital Universitario La Princesa, IIS-Princesa, 28006 Madrid, Spain; scastas@gmail.com

**Keywords:** latent tuberculosis infection, immune-mediated inflammatory diseases (IMIDs), polymedication associations, hepatotoxicity, isoniazid, methotrexate, DMARDs

## Abstract

**Background/Objectives**: Patients with rheumatic immune-mediated inflammatory diseases (R-IMID) require latent tuberculosis infection screening and, in case of positivity, chemoprophylaxis. Isoniazid INH remains the standard regimen, but hepatotoxicity is an underrecognized concern. To describe the characteristics of R-IMID patients developing hepatotoxicity during INH therapy and identify potential risk factors through clinical analysis and literature review. **Methods**: Retrospective study of 64 R-IMID who developed hepatotoxicity with INH. Mean age was 53.4 ± 10.5 years; 70.3% female. Diagnoses included spondyloarthritis/psoriatic arthritis (56.3%), rheumatoid arthritis (32.8%), systemic sclerosis (4.7%), connective tissue diseases (4.7%), and other IMIDs (3.2%). All patients showed ≥ 2 × upper limit of normality (ULN) liver enzyme elevation, 34.4% ≥ 3 ULN, 20.3% ≥ 4 ULN. Literature review (19 studies) revealed INH-related hepatotoxicity rates of 1–41%, exacerbated by concurrent methotrexate, sulfasalazine, TNF inhibitors, and prior drug-induced liver injury. **Results**: Hepatotoxicity was frequent when INH was combined with other hepatotoxic drugs, especially methotrexate. **Conclusions**: INH prophylaxis in R-IMID patients carries substantial hepatotoxic risk. Careful hepatic monitoring and individualized risk stratification are essential to prevent liver injury in immunosuppressed populations.

## 1. Introduction

Tuberculosis (TB) remains one of the most common and lethal infectious diseases worldwide. Although its global incidence has declined in recent decades, more than 10 million new cases and over 1 million deaths still occur annually, particularly among immune suppressed individuals [1,2].

Tuberculosis is caused by a bacteria with unique microbiological characteristics that require multidrug therapy to ensure complete eradication. This becomes particularly challenging in the context of contacts exposed to multidrug-resistant tuberculosis (MDR-TB). According to the World Health Organization, MDR-TB accounts for approximately 3.2% of new cases and 16% of previously treated cases. These epidemiological data underscore the complexity of selecting an appropriate LTBI chemoprophylaxis regimen in contacts of MDR-TB, where treatment choices depend largely on the resistance profile of the index case.

Latent tuberculosis infection (LTBI) refers to a state where *Mycobacterium tuberculosis* persists in an inactive form, with potential for reactivation during immunosuppressed states. In low-endemic countries such as Spain, LTBI remains the primary reservoir for active TB.

Patients with immune-mediated inflammatory diseases constitute a highly heterogeneous group, including rheumatoid arthritis, psoriatic arthritis, ankylosing spondylitis, and various connective-tissue disorders. Although each condition has distinct immunopathological mechanisms, many share common clinical features such as arthritis, myalgia, and systemic manifestations that may affect multiple organs and potentially lead to functional impairment. Because of both the underlying immune dysregulation and the immunosuppressive treatments these patients response to infections, particularly Mycobacterium tuberculosis, may vary considerably. It is plausible that these disease specific characteristics influence not only the prevalence of latent infection but also the risk and presentation of active TB.

This epidemiological pattern also accounts for the high proportion of women in our study population, consistent with the known female predominance in many rheumatic IMIDs.

This is important not only because LTBI has a high prevalence among patients with IMIDs, but also because these individuals are particularly vulnerable to reactivation. Their increased risk is not solely attributable to the initiation of tumor necrosis factor inhibitors (TNFi); but also because the underlying immune dysregulation inherent to the disease itself further predisposes them to TB reactivation [2,3].

Guidelines from the Centers for Disease Control and Prevention (CDC), European League Against Rheumatism (EULAR), and Spanish Society of Pulmonology and Thoracic Surgery (SEPAR) recommend universal LTBI screening prior to immunosuppressive therapy and advise against initiation if LTBI remains untreated. Screening is usually performed via tuberculin skin test (TST) and interferon-gamma release assay (IGRA), with chemoprophylaxis recommended if results are positive [3].

Most of the time, we assume that patients who initiate biologic therapy have not had recent exposure to active TB. For this reason, the standard LTBI regimen, isoniazid or rifampicin, remains the most widely used option, irrespective of previous exposure status. However, recently updated guidelines suggest that, depending on the type of TB contact and the resistance pattern involved, alternative initial regimens such as rifampicin, or even levofloxacin or moxifloxacin when rifampicin is not available, should be considered.

Common LTBI regimens include isoniazid (INH) for 6–9 months, rifampicin (RIF) for 4 months in cases of INH intolerance, or weekly rifapentine plus INH for 12 weeks, though this latter regimen is less frequently used in Europe [4,5,6].

It is important to notice that patients with prior TB treatment may experience two potential clinical scenarios: (1) true reactivation of latent infection or (2) reinfection following new exposure. These outcomes depend not only on epidemiological context but also on the patient’s immunological status. This highlights the clinical relevance of evaluating which chemoprophylaxis regimen is most appropriate for an individual patient, beyond the conventional single-drug approach with isoniazid.

Although generally well-tolerated, these regimens may cause hepatotoxicity in up to 28% of patients [6]. INH, the most widely used agent, inhibits mycolic acid synthesis and is effective against intracellular and extracellular bacilli [7], with neuropathy and drug-induced liver injury (DILI) as the main associated toxicities.

INH-induced hepatotoxicity is idiosyncratic, dose-independent, and may lead to acute liver failure or death [8]. Initially attributed to metabolic idiosyncrasy via acetyl hydrazine [9], later studies suggest that INH itself may produce reactive metabolites that trigger immune-mediated hepatic injury. In fact, two clinical phenotypes have been described: a self-limited, mild form and a severe variant associated with anti-cytochrome P450 autoantibodies, potentially causing progressive liver damage or death [10].

Several disease-modifying antirheumatic drugs (DMARDs), including hydroxychloroquine (HCQ), methotrexate (MTX), leflunomide (LEF), and sulfasalazine (SSZ), also may cause hepatotoxicity potential [11,12,13]. MTX disrupts folate metabolism, lowers antioxidant enzyme levels (e.g., S-adenosylmethionine [SAM]), induces oxidative stress via reactive oxygen species (ROS), and promotes proinflammatory cytokine activity through methylenetetrahydrofolate reductase (MTHFR) inhibition [11].

Risk factors for DILI include genetic susceptibility, age > 35 years, female sex, alcohol consumption, previous liver disease, and comorbid conditions such as human immunodeficiency virus (HIV), diabetes, hepatic steatosis, TB, or cancer [12]. Although not fully understood, the hepatotoxic risk associated with INH following DMARD-related DILI remains unclear and has been rarely reported; however, clinical practice often involves immunosuppression shortly after completing LTBI chemoprophylaxis, raising concerns about cumulative or synergistic liver injury [13,14].

Considering these factors, the objectives of this study were to identify the LTBI chemoprophylaxis regimens used in patients with R-IMIDs, estimate the incidence and risk factors for hepatotoxicity and other adverse events, and evaluate the risk of hepatic toxicity during INH and RIF administration in patients with prior DMARD-induced hepatotoxicity.

## 2. Materials and Methods

### 2.1. Patients and Study Design

A retrospective observational study was conducted at a tertiary university hospital in northern Spain between January 2016 and December 2020.

We included all patients with a diagnosis of R-IMID and a positive LTBI test. This was defined by a positive tuberculin skin test (TST) and/or interferon-gamma release assay (IGRA) (QuantiFERON-TB Gold Plus), marketed by the company QIAGEN, whose headquarters are in Venlo, The Netherlands. For those who received chemoprophylaxis prior to biological therapy (BT). Chemoprophylactic regimens included: (a) INH 5 mg/kg/day (maximum 300 mg) with vitamin B6 for 9 months; (b) RIF 10 mg/kg/day (maximum 600 mg) for 4 months; and (c) Levofloxacin 500 mg/day for 6–9 months. All patients underwent laboratory monitoring, including liver enzyme assessment at the first and third month, and thereafter at individualized intervals based on clinical judgment.

We also included consecutive patients diagnosed with R-IMID who developed hepatotoxicity with INH following DMARD treatment between 2016 and 2020. Hepatotoxicity was defined as ALT and/or AST elevation ≥2× the upper limit of normality (ULN) after treatment initiation. Data retrieved from patient records included age, sex, R-IMID diagnosis, liver enzyme elevation over baseline, and treatments received.

### 2.2. Data Collection

Data were extracted from electronic clinical records using a structured protocol specifically developed for this study to ensure uniformity and reproducibility of data collection. All variables of interest, including demographic characteristics, clinical history, immunosuppressive treatments, adverse events, and serial laboratory parameters were systematically recorded in predefined fields to minimize variability in interpretation.

Data abstraction was performed independently by two trained reviewers, who were blinded to each other’s evaluations. Any inconsistencies or discrepancies between reviewers were resolved through consensus after re-examining the original clinical documentation, thereby ensuring accuracy and internal validity. The final dataset was stored in a secure, password-protected electronic database with restricted access limited to authorized study personnel. To minimize transcription errors, double data-entry procedures were implemented, and periodic quality-control audits were carried out during the study period to ensure completeness, internal consistency, and adherence to the predefined data-collection protocol.

This study was conducted in accordance with the ethical principles of the Declaration of Helsinki and complies with national regulations regarding biomedical research and data protection. Ethical approval was granted by the Clinical Research Ethics Committee of Hospital Universitario Marqués de Valdecilla (Santander, Spain) (Protocol Number 2020.486), approved on 1 January 2016. Informed consent procedures followed institutional guidelines; given the retrospective design and anonymization of all patient data, the requirement of consent was waived by the Ethics Committee.

### 2.3. Statistical Analysis

Descriptive statistics were used to characterize demographic, clinical, and therapeutic variables. Categorical variables were expressed as absolute counts and relative frequencies, whereas continuous variables were summarized using means and standard deviations or medians and interquartile ranges, depending on distribution. Liver enzyme levels (ALT and AST) were examined both as continuous measures and according to clinically relevant categorical thresholds (≥2×, ≥3×, and ≥4× ULN), in line with internationally accepted definitions of hepatotoxicity.

**Figure 1:**
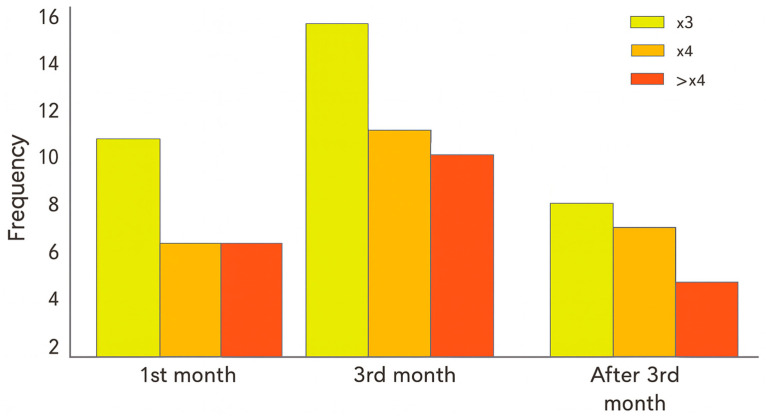
Liver enzyme elevation over baseline in patients with rheumatic immune-mediated inflammatory diseases receiving isoniazid for latent tuberculosis infection chemoprophylaxis.

**Figure 2:**
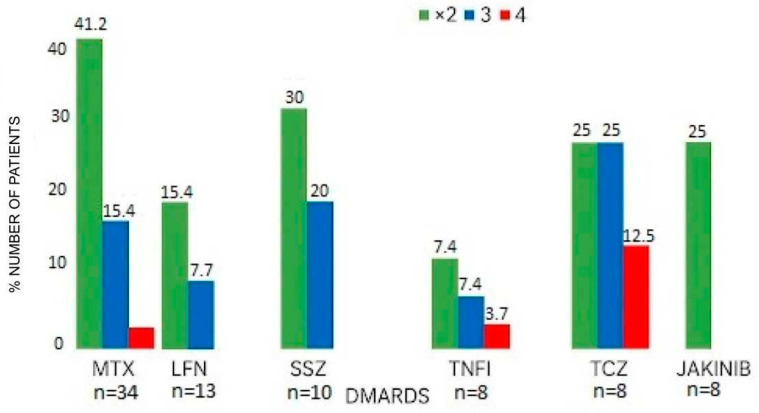
Hepatotoxicity with isoniazid after previous hepatic injury with DMARDS.

Adverse events, including any degree of liver enzyme elevation, were recorded and summarized at predefined time points (month 1, month 3, and beyond month 3), as detailed in Table 1. Patterns of hepatotoxicity were further explored across treatment subgroups (MTX, LEF, SSZ, TNFi, tocilizumab, and JAK inhibitors), and their temporal trajectories are presented in Figure 1 and Figure 2. The normality of quantitative variables was assessed using the Kolmogorov–Smirnov test. In cases where assumptions for parametric testing were not met, results were summarized using non-parametric descriptors. Missing data were addressed using complete-case analysis, given the low proportion of incomplete observations and the retrospective design of the study.

All statistical analyses were conducted using IBM SPSS Statistics software package, v.25. A two-tailed *p*-value of <0.05 was considered indicative of statistical significance.

### 2.4. Literature Review

Additionally, a literature review was conducted in July 2024 to identify studies on: (a) hepatotoxicity induced by INH in patients with LTBI and R-IMID; and (b) hepatotoxicity related to DMARDs to explore possible associations. The search aimed to estimate INH-related hepatotoxicity prevalence and its relationship to DMARD-related reactions.

We searched PubMed, Embase, the Cochrane Library and others from 1 January 2010, to 31 July 2024, without language restrictions, using the following keywords: (“latent tuberculosis” OR “latent tuberculosis infection” OR “LTBI”) AND (“prophylaxis” OR “isoniazid” OR “chemoprophylaxis” OR “rifampicin” OR “rifampin” OR “levofloxacin”) AND (“adverse reaction” OR “adverse effect” OR “DILI” OR “metabolic idiosyncrasy” OR “hepatotoxicity”) AND (“epidemiology”) AND (“rheumatic-IMID” OR “rheumatic inflammatory disease” OR “rheumatoid arthritis” OR “systemic lupus erythematosus” OR “Behçet’s disease”) AND (“hepatotoxicity related INH and DMARDs” OR “INH and methotrexate”).

Meta-analyses, reviews, cost-effectiveness studies, animal studies, and articles involving patients with active or presumed TB were excluded. According to this search strategy, nineteen articles met inclusion criteria, focusing on patients treated for LTBI and related adverse events in high-risk populations.

## 3. Results

### 3.1. Baseline Characteristics

Of 7218 screened patients with R-IMIDs, 240 received LTBI chemoprophylaxis: 232 with INH and 8 with RIF. Among them, 64 patients (27.6%) developed hepatotoxicity attributed to INH and were included in the analysis. Most were women (70.3%), with a mean age of 53.4 ± 10.5 years. Pre-existing liver disease was documented in 18.8% of cases, and six of these patients showed enzyme elevations during INH therapy.

Chronic alcohol consumption, defined as >210 g/week for men and >140 g/week for women. Only 6.7% of patients who developed hepatotoxicity met criteria for chronic alcohol use.

Underlying diagnoses included axial spondyloarthritis/psoriatic arthritis (56.3%), rheumatoid arthritis (RA) (32.8%), connective tissue diseases (CTDs) (4.7%), and other inflammatory conditions (3.2%). Most of them (81%) were scheduled to initiate TNFi therapy; others were treated with tocilizumab, abatacept, or alternative DMARDs (Table 1).

### 3.2. Anti-Tuberculosis Chemoprophylaxis

INH was prescribed in 232 patients (96.7%), while 8 (3.3%) received RIF. A subgroup analysis examined hepatotoxicity risk associated with prior DMARD exposure, with MTX, HCQ, LEF, and SSZ as the most frequently used drugs (Table 2).

### 3.3. Adverse Events

DMARD-related hepatotoxicity occurred in 35.2% of previously exposed patients. INH was also associated with peripheral neuropathy (1.25%), managed with pyridoxine, and mild gastrointestinal manifestations (3.3%). RIF showed fewer adverse events; only one case involved mild hepatic dysfunction.

Although the definition of acute liver failure may vary between the U.S. and Europe, it generally requires coagulopathy (INR > 1.7), bilirubin elevation (>3 mg/dL), prolonged jaundice (>3 days), and progression toward adverse outcomes such as hepatic encephalopathy, death, or need for liver transplantation. None of our patients developed acute liver failure or required hospitalization; all cases were managed on an outpatient basis. Most cases met criteria for mild to moderate hepatotoxicity, defined as ALT/AST elevations as defined as ALT/AST elevations <4× ULN.

### 3.4. Treatment Discontinuation

INH was discontinued in 10.3% of exposed patients: 17 due to hepatotoxicity, five due to gastrointestinal intolerance, and two for nonadherence. RIF was withdrawn in only one patient due to mild transaminase elevation.

### 3.5. Subgroup Analysis: Hepatotoxicity and DMARDs

Among the 64 patients with INH-induced hepatotoxicity, 34 had previously received MTX. Of these, 35.3% experienced MTX-related liver injury: eight required dose reductions and four MTX discontinuation. Peak transaminase elevations appeared approximately in the third month of INH therapy (Figure 1 and Figure 2).

### 3.6. Literature Review Data

**Figure 3:**
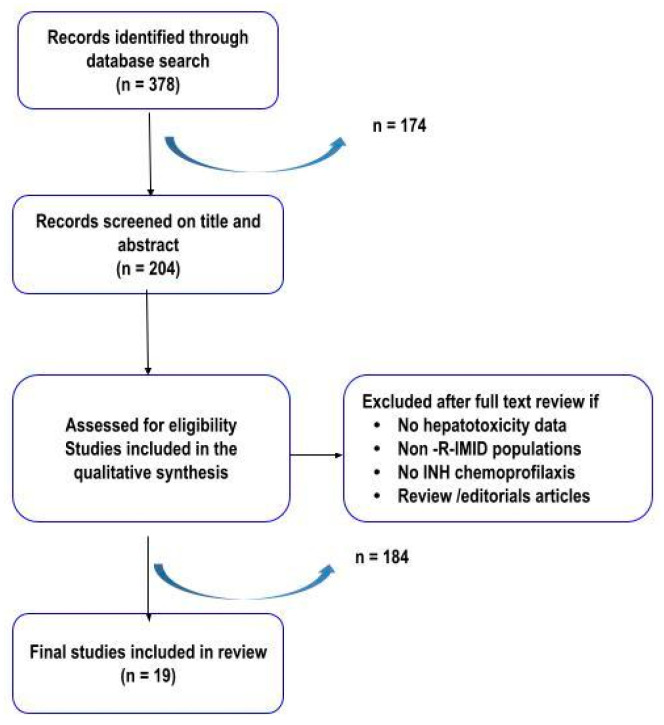
Flow chart of the systematic literature review process.

The literature review (Figure 3) supported the clinical link between INH-related hepatotoxicity and prior or concurrent DMARD therapy, particularly MTX. Hepatotoxicity ranged from asymptomatic transaminase elevations to significant liver injury requiring therapeutic intervention.

From 378 records, 19 studies met inclusion criteria after systematic screening. These included randomized trials, cohort studies, registries, abstracts, and consensus documents. Table 3 provides a summary of reported hepatotoxicity rates, which ranged from 1% to 41% among the included studies [15,16,17,18,19,20,21,22,23,24,25,26,27,28,29,30,31,32,33] with higher risk associated with MTX, SSZ, TNFi, and use of polymedication. Examples include the study by Sung et al., which reported that INH treatment for LTBI in patients with RA initiating TNF-inhibitors was associated with an increased occurrence of liver function tests (LFT) abnormalities [15]. In contrast, the study by Cansu et al. found that INH chemoprophylaxis was generally well tolerated in rheumatic patients receiving DMARDs, with a comparable rate of hepatotoxicity (*n* = 2, 3.3% vs. *n* = 1, 3.8%; *p* = 0.85) [16].

Unfortunately, the literature exploring this specific association is very limited. Few studies have evaluated whether hepatotoxicity induced by conventional DMARDs increases susceptibility to INH induced hepatotoxicity. Although combination therapy with DMARDs and LTBI prophylaxis is generally considered safe, isolated reports describe adverse events [21,22,30]. To this date, however, no published evidence demonstrates that patients who develop MTX induced hepatotoxicity are more likely to develop INH induced hepatotoxicity. Only one study did describe an increased risk of hepatotoxicity in patients receiving anti-TNF therapy in conjunction with DMARDs, but this does not specifically address the MTX–INH interaction [32].

## 4. Discussion

Our findings underscore the clinical relevance of INH-induced hepatotoxicity in patients with R-IMIDs undergoing LTBI chemoprophylaxis prior to BT. Although INH remains the preferred chemoprophylactic agent, its hepatic safety profile requires closer evaluation, especially in immunosuppressed individuals previously treated with DMARDs. In this cohort, 27.5% of patients receiving INH developed liver enzyme elevations. Most cases were mild and reversible, though five patients required discontinuation. This incidence exceeds prior estimates and reinforces current recommendations for liver function monitoring during the initial three months of treatment.

Although prescribed in only 3.3% of cases, RIF showed a more favorable hepatic profile, with only one case of mild transaminase elevation. It is important to emphasize that the number of patients receiving rifampicin was small and therefore this study does not allow reliable comparative interpretation. Nevertheless, the available literature suggests that rifampicin remains a safe alternative in this population.

These findings align with previous evidence suggesting a lower risk of hepatotoxicity with RIF compared to INH [34]. However, clinicians must weigh its enzyme-inducing potential and pharmacologic interactions in patients receiving multiple immunosuppressive therapies. Its hepatic tolerability profile suggests it may represent a safer alternative in patients with prior DMARD-related liver injury. RIF should be considered in patients with prior MTX-related liver toxicity, given the observed recurrence rate of nearly 50%.

A key observation was the increased incidence of hepatotoxicity in patients previously exposed to DMARDs, especially MTX. Among those with INH-induced liver injury, 35.3% had a documented history of MTX-related hepatotoxicity. This recurrence rate suggests a clinically relevant overlap in hepatotoxic mechanisms between MTX and INH, including mitochondrial dysfunction, oxidative stress, and impaired hepatic repair [35].

Additional risk factors, including alcohol consumption, obesity, and ageing, were prevalent and likely contributed to hepatic vulnerability [36,37,38]. Although patients with known liver disease were excluded, subclinical hepatic compromise may still predispose individuals to additive injury from immunosuppressant drugs [39].

Current evidence suggests that lifestyle factors, comorbidities, genetic susceptibility, and prior DMARD-related liver toxicity increase the risk of hepatotoxicity following initiation of INH prophylaxis. Nonetheless, heterogeneity across reviewed studies limits direct comparisons. While some studies suggest that combining these drugs may be safe, others advise against it, particularly in patients with prior DMARD-induced hepatotoxicity. Despite potential confounders, our data suggest that patients with prior MTX-induced DILI are at increased risk of hepatotoxicity with INH. This association, which emerges as a key finding in our analysis, has not been clearly reported elsewhere.

Most hepatotoxic events occurred in the third month of INH prophylaxis. This late temporal pattern is consistent with mechanisms such as cumulative metabolic stress, oxidative injury, and synergy with concomitant DMARDs. However early toxicity is related to pre-existing liver abnormalities and risk factors such as alcohol consumption, metabolic syndrome, or idiosyncratic susceptibility. Highly consistent with our findings.

These findings carry practical implications: patients with prior MTX-induced liver injury appear at high risk for INH-related hepatotoxicity across varying degrees of severity. In such cases, alternative prophylactic agents like RIF may help avoid treatment interruption, reduce monitoring burden, and prevent delays in disease control. Management may include enhanced monitoring, temporary interruption, or permanent discontinuation of INH.

This may open several avenues for future research, including the evaluation of whether a shared pathophysiological mechanism exists between these two drugs and whether this relationship is specific to patients with IMID. It also highlights the need to compare safer therapeutic alternatives tailored to the treatments commonly used in this population, including regimens based on fluoroquinolones. Additionally, the development of clinical risk-score models incorporating relevant variables could help predict which patients are more likely to develop hepatotoxicity.

Strengths of this study include a well-defined R-IMID cohort, standardized monitoring protocols, and integration of a targeted literature review to enhance external validity. As shown in Table 3 several reviewed studies reported elevated hepatotoxicity risk in patients receiving INH and hepatotoxic immunosuppressant agents, including MTX, SSZ, and TNFi [40].

The limitations of our study include the retrospective, observational design, lack of histologic confirmation of hepatotoxicity in most of the patients, and limited representation of other alternative prophylactic agents such as levofloxacin or extended RIF-based regimens. The absence of pharmacogenetic stratification, such as NAT2 genotyping [41], also constrains individualized risk assessment.

It is important to acknowledge a relevant limitation in the analysis and interpretation of our data. In most instances, patients who developed mild hepatotoxicity did not require discontinuation of therapy; instead, they were managed with closer monitoring of liver function tests. However, for analytical purposes, these episodes were still recorded and classified as mild hepatotoxicity cases. This should be taken into account, as it may influence the interpretation of the severity and the actual clinical impact of the hepatic abnormalities observed.

## 5. Conclusions

In summary, isoniazid remains the first-line agent for LTBI prevention in patients with R-IMIDs; however, hepatotoxicity affects more than one quarter of exposed individuals, particularly those with a history of MTX-induced liver injury, an association that had not been clearly described until now. Given that nearly half of these patients experienced recurrent hepatotoxicity with INH, our findings suggest that rifampicin or other regimens may represent better-tolerated alternatives in this subgroup. Nevertheless, the small number of patients treated with rifampicin in our cohort precludes robust comparative conclusions. Overall, these results support the adoption of individualized prophylactic strategies, highlight the need for close hepatic monitoring to enhance safety and long-term outcomes, and raise the possibility of a shared pathophysiological mechanism underlying hepatotoxicity in MTX and INH in patients with IMID.

## Figures and Tables

**Table 1:** Adverse events with isoniazid at months 1, 3, and after month 3.

	Month 1 (*n* = 232)			Month 3 (*n* = 222)			After Month 3 (*n* = 214)		
Adverse Events, *n* (%)	Total	Switching INH	Stop INH	Total	Switching INH	Stop INH	Total	Switching INH	Stop NH
Hepatotoxicity	38 (16.4)	6 (2.6)	0	33 (14.9)	5 (2.3)	2 (0.9)	16 (7.5)	2 (0.9)	0
Gastrointestinal side effects	4 (1.3)	3 (1.3)	1 (0.4)	3 (1.5)	0	3 (1.5)	2 (0.9)	0	1 (0.5)
Cutaneous toxicity	2 (0.9)	1 (0.4)	0	0	0	0	0	0	0
Dizziness	1 (0.4)	1 (0.4)	0	0	0	0	0	0	0
Total	44 (19.0)	11 (4.7)	1 (0.4)	36 (16.2)	5 (2.3)	5 (2.2)	18 (8.4)	2 (0.9)	1 (0.5)

*n*: number.

**Table 2:** Main characteristics of 64 patients with rheumatic IMID who developed hepatotoxicity during isoniazid therapy.

Variables	Patients (*n* = 64)
Age (years), mean ± SD	53.4 ± 10.5
Sex (women), *n* (%)	45 (70.3)
Rheumatic-IMID diagnosis, *n* (%)	
SpA/PsA	36 (56.3)
RA	21 (32.8)
SSc	3 (4.7)
CTD (SLE, Myositis, etc.)	3 (4.7)
Other (vasculitis, sarcoidosis)	2 (3.2)
Liver enzyme elevation over baseline (INH) ×2	64 (100)
×3	22 (34.4)
×4 or higher	13 (20.3)
csDMARDs, *n* (%)	
MTX	34 (53.1)
HCQ	15 (23.4)
LEF	13 (20.3)
SSZ	10 (15.6)
bDMARDs, *n* (%)	47 (73.4)
Targeted synthetic DMARDs (Jakinibs), *n* (%)	8 (12.5)

Abbreviations (in alphabetical order): ALT, alanine aminotransferase; AST, aspartate aminotransferase; bDMARD, biological disease-modifying antirheumatic drugs; csDMARD, conventional synthetic disease-modifying antirheumatic drugs; CTD, connective tissue diseases; HCQ, hydroxychloroquine; IMID, immune-mediated inflammatory diseases; INH, isoniazid; Jakinibs, Janus kinase inhibitors; LEF, leflunomide; MTX, methotrexate; *n*, number; PsA, psoriatic arthritis; RA, rheumatoid arthritis; rheumatic-IMID, rheumatic immune-mediated inflammatory diseases; SLE, systemic lupus erythematosus; SSc, systemic sclerosis; SD, standard deviation; SpA, spondyloarthritis; SSZ, sulfasalazine; ULN, upper limit of normality; ×2, 3, 4, multiplied by 2, 3 or 4.

**Table 3:** Summary of published studies on isoniazid-related hepatotoxicity in patients with rheumatic-IMID.

Author (Year) (Ref. in Text)	Study Design	Population	Isoniazid Use	Details
Sung et al. (2018) [15]	Observational	RA patients receiving TNFi	Yes	INH associated with LFT abnormalities after DMARD initiation
Cansu et al. (2014) [16]	Observational	RA patients receiving TNFi and DMARDs	Yes	No significant increase in hepatotoxicity with INH
Mor et al. (2008) [17]	Observational	RA patients receiving MTX	Yes	INH + MTX showed acceptable hepatic safety
Xie et al. (2009) [18]	Observational	Chinese RA patients receiving MTX	Yes	INH prophylaxis during MTX; tolerability evaluated
Valls & Ena (2015) [19]	Prospective	Rheumatic patients prior to TNFi initiation	Yes	Short-course LTBI regimens tested
Cataño & Morales (2015) [20]	Follow-up study	Patients receiving biologic therapy	Yes	INH chemoprophylaxis prior to biologics
Bourré-Tessier et al. (2014) [21]	Observational	RA patients receiving DMARDs/biologics	Yes	Elevated LFTs observed with INH
Haroon et al. (2012) [22]	Observational	RA/psoriasis patients	Yes	30% intolerance rate to INH-based TB prophylaxis
Goletti et al. (2018) [23]	Narrative review	Rheumatic patients receiving biologics	Yes	Overview of TB preventive therapy. Tolerance to INH
Gómez-Reino et al. (2003) [24]	Registry study	Patients treated with TNFi	Yes	Risk of TB assessed in registry. INH appears to be safe
Tsai et al. (2012) [25]	Pooled clinical trials	Psoriasis patients receiving ustekinumab	Yes	LTBI patients received INH; treatment safe
Anton et al. (2019) [26]	Observational	Rheumatic diseases	Yes	Prevalence of LTBI; INH use described
Jha et al. (2025) [27]	Review	IMID patients	Yes	Screening and management of LTBI before therapies. INH safe.
Kaptan et al. (2021) [28]	Observational	Patients receiving TNFi	Yes	TB despite LTBI screening/treatment
Hanta et al. (2007) [29]	Observational	Rheumatic patients receiving TNFi	Yes	INH in 86 patients; tolerability described
Cataño & Morales (2016) [30]	Observational	Psoriasis on biologics	Yes	INH toxicity and TB cases observed
Yun et al. (2007) [31]	Observational	Arthritis patients receiving TNFi	Yes	LTBI diagnosis and INH treatment described to be safe
Lai et al. (2021) [32]	Observational	RA patients receiving TNFi	Yes	9-month INH prophylaxis associated with severe hepatitis
Hazlewood et al. (2013) [33]	Decision analysis	Elderly RA patients	Yes	LTBI prophylaxis prior to TNFi model

Abbreviations (in alphabetical order): anti-TNF: tumor necrosis factor inhibitors; AS: ankylosing spondylitis; DMARDs: disease-modifying anti-rheumatic drugs; HCQ: hydroxychloroquine; INH: isoniazid; R-IMID: rheumatic immune-mediated inflammatory diseases; LFTs: liver function tests; LTBI: latent tuberculosis infection; MTX: methotrexate; OR: odds ratio; RA: rheumatoid arthritis; SSZ: sulfasalazine; ULN: upper limit of normality.

## Data Availability

Data is available upon reasonable request by any qualified researchers who engage in rigorous, independent scientific research, and will be provided following review and approval of a research proposal and Statistical Analysis Plan (SAP) and execution of a Data Sharing Agreement (DSA). All data relevant to the study are included in the article.

## Abbreviations

The following abbreviations are used in this manuscript:

AS	Ankylosing spondylitis
PsA	Psoriatic arthritis
BD	Behçet’s disease
BT	biological therapy
CDC	Centers for Disease Control and Prevention
CTDs	connective tissue diseases
DILI	drug-induced liver injury
DMARDs	disease-modifying antirheumatic drugs
EULAR	European League Against Rheumatism
HCQ	Hydroxychloroquine
HIV	Human immunodeficiency virus
IGRA	Interferon-gamma release assay
INH	Isoniazid
JAKi	Janus kinase inhibitors
LEF	Leflunomide
LFT	liver function tests
LTBI	Latent tuberculosis infection
MTHFR	methylenetetrahydrofolate reductase
PPD	Purified protein derivative
RA	Rheumatoid arthritis
Rheumatic-IMID	Rheumatic immune-mediated inflammatory diseases
RIF	Rifampicin
SAM	S-adenosylmethionine
SEPAR	Spanish Society of Pneumology and Thoracic Surgery
SLE	Systemic lupus erythematosus
SpA	Spondyloarthritis
SSZ	Sulfasalazine
TNFi	Tumor necrosis factor inhibitors
TCZ	Tocilizumab
TST	Tuberculin skin test
ULN	upper limit of normality
WHO	World Health Organization

## References

[1] World Health Organization (2022). WHO Unified Guidelines on Tuberculosis: Preventive Treatment of Tuberculosis.

[2] Kiazyk S., Ball T.B. (2017). Latent tuberculosis infection: An overview. Can. Commun. Dis. Rep..

[3] Osorio-Chávez J.S., Martínez-López D., Álvarez-Reguera C., Portilla V., Cifrián J.M., Castañeda S., Ferraz-Amaro I., Blanco R. (2024). Epidemiology of latent tuberculosis in rheumatic immune-mediated inflammatory diseases: Study of 1117 patients and descriptive literature review. J. Clin. Med..

[4] Sterling T.R., Njie G., Zenner D., Cohn D.L., Reves R., Ahmed A., Menzies D., Horsburgh C.R., Crane C.M., Burgos M. (2020). Guidelines for the treatment of latent tuberculosis infection: Recommendations from the National Tuberculosis Controllers Association and CDC, 2020. MMWR Recomm. Rep..

[5] Fragoulis G.E., Nikiphorou E., Dey M., Zhao S.S., Courvoisier D.S., Arnaud L., Atzeni F., Behrens G.M., Bijlsma J.W., Böhm P. (2023). 2022 EULAR recommendations for screening and prophylaxis of chronic and opportunistic infections in adults with autoimmune inflammatory rheumatic diseases. Ann. Rheum. Dis..

[6] González-Martín J., García-García J.M., Anibarro L., Vidal R., Esteban J., Blanquer R., Moreno S., Ruiz-Manzano J. (2010). Documento de consenso sobre diagnóstico, tratamiento y prevención de la tuberculosis. Arch. Bronconeumol..

[7] Schroeder E.K., de Souza N., Santos D.S., Blanchard J.S., Basso L.A. (2002). Drugs that inhibit mycolic acid biosynthesis in Mycobacterium tuberculosis. Curr. Pharm. Biotechnol..

[8] Metushi I., Uetrecht J., Phillips E. (2016). Mechanism of isoniazid-induced hepatotoxicity: Then and now. Br. J. Clin. Pharmacol..

[9] Jee A., Sernoskie S.C., Uetrecht J. (2021). Idiosyncratic drug-induced liver injury: Mechanistic and clinical challenges. Int. J. Mol. Sci..

[10] Metushi I.G., Sanders C., Lee W.M., Uetrecht J. (2014). Detection of anti-isoniazid and anti-cytochrome P450 antibodies in patients with isoniazid-induced liver failure. Hepatology.

[11] Wang Y.C., Chiang E.P. (2012). Low-dose methotrexate inhibits methionine S-adenosyltransferase in vitro and in vivo. Mol. Med..

[12] Chalasani N., Björnsson E. (2010). Risk factors for idiosyncratic drug-induced liver injury. Gastroenterology.

[13] Vanhoof J., Landewe S., Van Wijngaerden E., Geusens P. (2003). High incidence of hepatotoxicity of isoniazid treatment for tuberculosis chemoprophylaxis in patients with rheumatoid arthritis treated with methotrexate or sulfasalazine and anti-tumour necrosis factor inhibitors. Ann. Rheum. Dis..

[14] Russom M., Jeannetot D.Y.B., Berhane A., Woldu H.G., Stricker B.H., Verhamme K.M.C. (2023). Liver injury following isoniazid preventive therapy in HIV patients attending Halibet National Referral Hospital, Eritrea: A prospective cohort study. Drugs Real World Outcomes.

[15] Sung Y.K., Cho S.K., Kim D., Won S., Choi C.B., Kim T.H., Jun J.B., Yoo D.H., Bae S.C. (2018). Isoniazid treatment for latent tuberculosis infection is tolerable for rheumatoid arthritis patients receiving tumor necrosis factor inhibitor therapy. Korean J. Intern. Med..

[16] Cansu D.Ü., Güncan S., Bilge N.Ş., Kaşifoğlu T., Korkmaz C. (2014). Does isoniazid chemoprophylaxis increase the frequency of hepatotoxicity in patients receiving anti-TNF-α agent with a disease-modifying antirheumatic drug?. Eur. J. Rheumatol..

[17] Mor A., Bingham C.O., Kishimoto M., Izmirly P.M., Greenberg J.D., Reddy S., Rosenthal P.B. (2008). Methotrexate combined with isoniazid treatment for latent tuberculosis is well tolerated in patients with rheumatoid arthritis: Experience from an urban arthritis clinic. Ann. Rheum. Dis..

[18] 18, Xie Q.B., Wen F.Q., Yin G. (2009). Isoniazid prophylaxis for pulmonary tuberculosis in Chinese patients with rheumatoid arthritis receiving long-term methotrexate therapy. J. Sichuan Univ. Med. Sci. Ed..

[19] Valls V., Ena J. (2015). Short-course treatment of latent tuberculosis infection in patients with rheumatic conditions proposed for anti-TNF therapy. Clin. Rheumatol..

[20] Cataño J.C., Morales M. (2015). Follow-up results of isoniazid chemoprophylaxis during biological therapy in Colombia. Rheumatol. Int..

[21] Bourré-Tessier J., Arino-Torregrosa M., Choquette D. (2014). Increased incidence of liver enzymes abnormalities in patients treated with isoniazid in combination with disease modifying and/or biologic agents. Clin. Rheumatol..

[22] Haroon M., Martin U., Devlin J. (2012). High incidence of intolerance to tuberculosis chemoprophylaxis. Rheumatol. Int..

[23] Goletti D., Petrone L., Ippolito G., Niccoli L., Nannini C., Cantini F. (2018). Preventive therapy for tuberculosis in rheumatological patients undergoing therapy with biological drugs. Expert Rev. Anti Infect. Ther..

[24] Gómez-Reino J.J., Carmona L., Carmona L., Valverde V.R., Mola E.M., Montero M.D. (2003). Risk of tuberculosis in patients treated with TNF antagonists: Observational registry. Arthritis Rheum..

[25] Tsai T.F., Ho V., Song M., Szapary P., Kato T., Wasfi Y., Li S., Shen Y., Leonardi C., on behalf of the PHOENIX 1, PHOENIX 2, ACCEPT, PEARL (2012). The safety of ustekinumab treatment in patients with moderate-to-severe psoriasis and latent tuberculosis infection. Br. J. Dermatol..

[26] Anton C., Machado F.D., Ramirez J.M.A., Bernardi R.M., Palominos P.E., Brenol C.V., Mello F.C.d.Q., Silva D.R. (2019). Latent tuberculosis infection in patients with rheumatic diseases. J. Bras. Pneumol..

[27] Jha D.K., Kakadiya R., Sharma A., Naidu S., De D., Sharma V. (2025). Assessment and management for latent tuberculosis before advanced therapies for immune-mediated inflammatory diseases: A comprehensive review. Autoimmun. Rev..

[28] Kaptan Y., Suner A., Taş M.N., Oksel F., Aksu K., Sayiner A. (2021). Tuberculosis despite latent infection screening and treatment in patients receiving TNF inhibitor therapy. Clin. Rheumatol..

[29] Hanta I., Ozbek S., Kuleci S., Sert M., Kocabas A. (2007). Isoniazid intervention for latent tuberculosis among 86 patients with rheumatologic disease administered with anti-TNFalpha. Clin. Rheumatol..

[30] Cataño J., Morales M. (2016). Isoniazid toxicity and TB development during biological therapy of patients with psoriasis in Colombia. J. Dermatol. Treat..

[31] Yun J.W., Lim S.Y., Suh G.Y., Chung M.P., Kim H., Kwon O.J., Cha H.S., Koh E.M., Koh W.J. (2007). Diagnosis and treatment of latent tuberculosis infection in arthritis patients treated with tumor necrosis factor antagonists in Korea. J. Korean Med. Sci..

[32] Lai E.C., Liang H.Y., Huang Y.C., Huang W.I., Chao P.H., Chen W.W., Weng M.Y. (2021). Association between 9-month isoniazid prophylaxis of latent tuberculosis and severe hepatitis in patients treated with TNF inhibitors. Sci. Rep..

[33] Hazlewood G.S., Naimark D., Gardam M., Bykerk V., Bombardier C. (2013). Prophylaxis for latent tuberculosis infection prior to anti–tumor necrosis factor therapy in low-risk elderly patients with rheumatoid arthritis: A decision analysis. Arthritis Care Res..

[34] Page K.R., Sifakis F., Montes de Oca R. (2006). Improved adherence and less toxicity with rifampin vs isoniazid for treatment of latent tuberculosis: A retrospective study. Arch. Intern. Med..

[35] Schmidt S., Messner C.J., Gaiser C., Hämmerli C., Suter-Dick L. (2022). Methotrexate-induced liver injury is associated with oxidative stress, impaired mitochondrial respiration, and endoplasmic reticulum stress in vitro. Int. J. Mol. Sci..

[36] Tiwari V., Shandily S., Albert J., Mishra V., Dikkatwar M., Singh R., Sah S.K., Chand S. (2025). Insights into medication-induced liver injury: Understanding and management strategies. Toxicol. Rep..

[37] Åberg F., Färkkilä M. (2020). Drinking and obesity: Alcoholic liver disease/nonalcoholic fatty livers disease interactions. Semin. Liver Dis..

[38] Humphreys J.H., Warner A., Costello R., Lunt M., Verstappen S.M.M., Dixon W.G. (2017). Quantifying the hepatotoxic risk of alcohol consumption in patients with rheumatoid arthritis taking methotrexate. Ann. Rheum. Dis..

[39] Ghabril M., Vuppalanchi R., Chalasani N. (2025). Drug-induced liver injury in patients with chronic liver disease. Liver Int..

[40] Park J.W., Curtis J.R., Lee H., Lee J.K., Song Y.W., Lee E.B. (2020). Risk-benefit analysis of isoniazid monotherapy to prevent tuberculosis in patients with rheumatic diseases exposed to prolonged, high-dose glucocorticoids. PLoS ONE.

[41] Richardson M., Kirkham J., Dwan K., Sloan D.J., Davies G., Jorgensen A.L. (2019). NAT2 variants and toxicity related to anti-tuberculosis agents: A systematic review and meta-analysis. Int. J. Tuberc. Lung Dis..

